# Supplementary material for: Quantum-coherent mixtures of causal relations
Source: Nat Commun. 2017 May 9;8:15149. doi: 10.1038/ncomms15149 (PMC5436107; doi:10.1038/ncomms15149)
Supplement: Supplementary Information — Supplementary Notes and Supplementary References. [file ncomms15149-s1.pdf]

SUPPLEMENTARY NOTES

Supplementary Note 1: Probabilistic mixtures of common-cause and cause-effect relations

As defined in the main text of the article, a causal map $\mathcal{E}_{CB|D}$ is said to physically realize a probabilistic mixture of cause-effect and common-cause relations if it is possible to express it as follows: there is a hidden classical control variable, J, which only influences B, such that for every value of J, either B depends only on D in the causal map or B depends only on its common cause with C. In this section, we discuss why this is the appropriate notion of probabilistic mixture to study. We also demonstrate that it implies that the causal map has the form

$$\mathcal{E}_{CB|D} = w\mathcal{E}_{B|D} \otimes \rho_C + (1-w)\rho_{CB} \otimes \mathrm{Tr}_D, \tag{1}$$

where $0 \leq w \leq 1$ and

$$\mathrm{Tr}_B \rho_{CB} = \rho_C. \tag{2}$$

We are here concerned with what sorts of probabilistic mixtures of causal structures are physically realizable. Note that a probabilistic mixture of alternatives is always physically realized by making the choice of alternatives depend causally on the value of a control variable that has been sampled from some probability distribution and for which the value is not observed. It follows that to be physically realizable, a probabilistic mixture of the elements of a set of causal maps, $\{\mathcal{E}^{(j)}_{CB|D}\}$, must have the form $\mathcal{E}_{CB|D} = \sum_j P(j)\mathcal{E}^{(j)}_{CB|D}$, where J denotes the hidden control variable and $P(j)$ is the probability that $J = j$.

What is critical to recognize is that the causal dependence of systems on the control variable cannot be treated abstractly but must instead be considered as part of the causal structure. One can then ask whether one can infer any constraints on the causal structure of the probabilistic mixture from the causal structure of the elements appearing in the mixture. We argue that there is indeed a very natural constraint:

> If all of the causal maps in a probabilistic mixture describe the same causal relation, then their mixture should describe this causal relation as well.

Note, first of all, that one particular implication of this constraint is that a causal map that is a probabilistic mixture of purely cause-effect maps should be purely cause-effect. We now demonstrate how this constraint implies a restriction on the sorts of probabilistic mixtures that can be physically realized.

To begin, we consider the possibility that the set of probabilistic mixtures of causal structures that are physically realizable is the full set of such mixtures. In this case, the causal maps corresponding to physically-realizable probabilistic mixtures of cause-effect and common-cause relations would be those that are a convex sum of causal maps each of which is purely cause-effect or purely common-cause, that is, those of the form

$$\mathcal{E}_{CB|D} = \sum_{j\in\mathcal{J}_1} P(j)\mathcal{E}^{(j)}_{B|D} \otimes \rho^{(j)}_C + \sum_{j\in\mathcal{J}_2} P(j)\rho^{(j)}_{CB} \otimes \mathrm{Tr}_D, \tag{3}$$

where the set of values of J are partitioned into two subsets, denoted $\mathcal{J}_1$ and $\mathcal{J}_2$, and P is a probability distribution thereon.

In order to realize such a causal map, the control variable J needs to have a causal influence on both B and C. Otherwise, we could not explain how the marginal states on B and on C both vary with the value of J. In this case, J acts as a common cause of B and C, and the causal structure of the overall causal map is that of Supplementary Fig. 1(a).

The alternative proposal, the one that we endorse here, is that the control variable J only has a causal influence on B. In this case, the causal structure of the overall causal map is that of Supplementary Fig. 1(b).

We now demonstrate that the assumption that J is a cause of both B and C violates the natural constraint articulated above. Consider first the implication of the constraint for probabilistic mixtures of purely cause-effect maps. If every map in the probabilistic mixture is purely cause-effect, then for all values j of J, $\mathcal{E}^{(j)}_{CB|D} = \mathcal{E}^{(j)}_{B|D} \otimes \rho^{(j)}_C$. The fact that J can influence B and C is encoded here in the fact that the marginal on C is j-dependent. But now consider the causal map associated to this probabilistic mixture. It is $\mathcal{E}_{CB|D} = \sum_j w_j \mathcal{E}^{(j)}_{B|D} \otimes \rho^{(j)}_C$. This is not a purely cause-effect map in general because, by definition, such maps must take the form of a tensor product of a map from D to B and a state on C. We can understand this by noting that the control variable acts as a common cause, so that the causal structure is that depicted in Supplementary Fig. 1(c), which is clearly not purely cause-effect. Thus

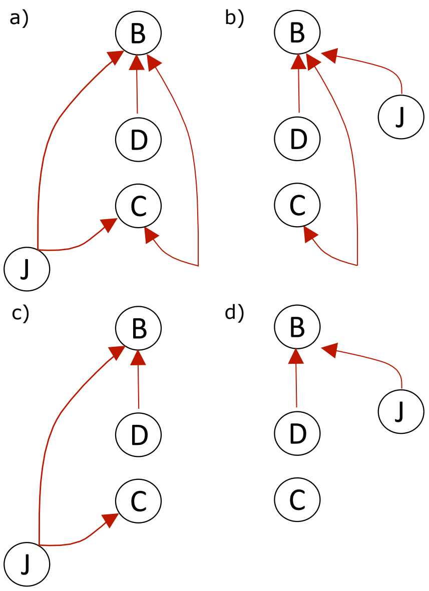

Supplementary Fig. 1. **a)** Causal relations between A (split into C and D) and B where the control variable J acts as a common cause for both B and C. **b)** We propose that a causal map represents a probabilistic mixture of common-cause and cause-effect if it is possible to achieve it by a hidden control variable J acting only on B. **c,d)** If one mixes only cause-effect relations, then the result, according to our constraint, should also be purely cause-effect. Again, this condition is not satisfied if the control variable J acts as a common cause of B and C, but it is if J influences only B.

if one demands that any physical realization of a probabilistic mixture of purely cause-effect maps should itself be purely cause-effect, the scheme just described does not in fact realize such a mixture.

On the other hand, if we demand that J can only influence B, as in Supplementary Fig. 1(d), then $\mathcal{E}_{CB|D} = \left(\sum_j \mathcal{E}^{(j)}_{B|D}\right) \otimes \rho_C$. Here the marginal on C is j-independent and therefore can be factorized out of the sum. This is a purely cause-effect map, and so the constraint is satisfied.

We now show how to prove that all physically-realizable probabilistic mixtures can be expressed as a probabilistic mixture of just two causal maps, one of which is purely cause-effect and the other of which is purely common-cause.

By our definition, it must be possible to partition the values of J into two subsets, denoted $\mathcal{J}_1$ and $\mathcal{J}_2$, where for $j \in \mathcal{J}_1$, B depends only on D, so that $\mathcal{E}^{(j)}_{CB|D} = \mathcal{E}^{(j)}_{B|D} \otimes \rho_C$, and where for $j \in \mathcal{J}_2$, B depends only on the common cause with C, so that $\mathcal{E}^{(j)}_{CB|D} = \rho^{(j)}_{CB} \otimes \mathrm{Tr}_D$. The fact that the control variable J is assumed to have no influence on C implies that for all values of J, the causal map $\mathcal{E}^{(j)}_{CB|D}$ must have the same marginal on C. This is why ρ_C has no dependence on j in the expression for $\mathcal{E}^{(j)}_{CB|D}$ when $j \in \mathcal{J}_1$. The lack of influence of J on C also implies that we must have

$$\mathrm{Tr}_B \rho^{(j)}_{CB} = \rho_C, \tag{4}$$

for all $j \in \mathcal{J}_2$.

The overall causal map is obtained by weighting the $\mathcal{E}^{(j)}_{CD|D}$ by the probability $P(j)$ of their occurrence, so that

$$\mathcal{E}_{CB|D} = \sum_{j\in\mathcal{J}_1} P(j)\mathcal{E}^{(j)}_{B|D} \otimes \rho_C + \sum_{j\in\mathcal{J}_2} P(j)\rho^{(j)}_{CB} \otimes \mathrm{Tr}_D.$$

Finally, defining

$$w \equiv \sum_{j\in\mathcal{J}_1} P(j),$$

and

$$\mathcal{E}_{B|D} \equiv \frac{1}{w}\sum_{j\in\mathcal{J}_1} P(j)\mathcal{E}^{(j)}_{B|D},$$

and

$$\rho_{CB} \equiv \frac{1}{1-w} \sum_{j \in \mathcal{J}_2} P(j) \rho_{CB}^{(j)}, \tag{5}$$

we obtain supplementary equation 1. Supplementary equation 5 together with supplementary equation 4 implies supplementary equation 2.

Supplementary Note 2: The Choi isomorphism and different classes of causal maps

We begin by introducing a useful tool for defining and characterizing causal maps that puts quantum channels, viz completely positive and trace-preserving (CPTP) maps, on an equal footing with bipartite quantum states. The Choi isomorphism [1] (see also [2]) establishes that completely positive maps from linear operators on the Hilbert space of A to linear operators on B, denoted $\mathcal{E}_{B|A} : \mathcal{L}(\mathcal{H}_A) \to \mathcal{L}(\mathcal{H}_B)$, are isomorphic to positive-semidefinite operators $\tau_{BA} \in \mathcal{H}_B \otimes \mathcal{H}_A$ given by

$$\tau_{BA} \equiv \left(\mathcal{E}_{B|A'} \otimes \mathbb{1}_A\right) \left(\left|\Phi^+\right\rangle \left\langle\Phi^+\right|_{A'A}\right). \tag{6}$$

Here, $|\Phi^+\rangle_{A'A} = \frac{1}{\sqrt{d}} \sum_{k=1}^{d} |k\rangle_{A'} |k\rangle_A$ denotes the symmetric, maximally entangled state between A and an ancilla, A', where d is the Hilbert space dimension of A and A'. (Different choices of $\{|k\rangle\}$ lead to different forms of the Choi state; we will fix a convention for our calculations below.) Note that the different subscript in $\mathcal{E}_{B|A'}$ indicates that the map is acting on input A' and taking it to output B, as shown in Supplementary Fig. 2. Since $|\Phi^+\rangle$ is normalized, τ_{BA} also has unit trace, making it a valid quantum state, if the map is trace-preserving. We refer to τ_{BA} as the Choi state of the map $\mathcal{E}_{B|A}$.

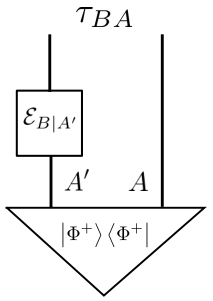

Supplementary Fig. 2. Operational interpretation of the Choi state τ_{BA}. It can be prepared by starting with the maximally entangled state $|\Phi^+\rangle$ on A and A' and applying the map to A'.

The isomorphism also allows us to express the effect of the map on a generic input in terms of its Choi state [3]: for any linear operator ρ_A on $\mathcal{H}_A$,

$$\begin{aligned}\mathcal{E}_{B|A}(\rho_A) &= d \ \mathrm{Tr}_A \left[(T_A \tau_{BA}) \mathbb{1}_B \otimes \rho_A\right] \\ &= d \ \mathrm{Tr}_A \left[\tau_{BA} \cdot (\mathbb{1}_B \otimes T_A \rho_A)\right].\end{aligned} \tag{7}$$

The transposition on A, denoted T_A, must be included in this expression in order for τ_{BA} to be a positive operator. It can be applied either to the input ρ_A or to the Choi state itself. The identity operator on B, which is formally required in order for us to multiply τ_{BA} and ρ_A, is often omitted in the following for brevity.

Example classes of causal maps

In the following section, we show how the circuits presented in Fig. realize examples of the causal maps $\mathcal{E}_{CB|D}$ in the classes CoH (hence PhysQ), ProbQ, ProbC, and PhysC, respectively. In each case, we begin with a specification

of the circuit elements, namely the state ρ_{CE}, which will be taken to be the maximally entangled state $|\Phi^+\rangle$ in all following cases, and the gate $\mathcal{E}_{BF|DE}$, and find the causal map via

$$\mathcal{E}_{CB|D}(\cdot) = \mathrm{Tr}_F \circ \mathcal{E}_{BF|DE}(\,\cdot\, \otimes \rho_{CE}). \tag{8}$$

We then derive the associated Choi state, $\tau_{CBD} \in \mathcal{L}(\mathcal{H}_C \otimes \mathcal{H}_B \otimes \mathcal{H}_D)$, which is given by

$$\begin{aligned}\tau_{CBD} &= (\mathcal{E}_{CB|D'} \otimes \mathcal{I}_D)\left(\left|\Phi^+\right\rangle\left\langle\Phi^+\right|_{D'D}\right)\\ &= \frac{1}{2}\sum_{j,k} \mathcal{E}_{CB|D'}\left(|j\rangle\langle k|_{D'}\right) \otimes |j\rangle\langle k|_D\,,\end{aligned} \tag{9}$$

and use the witnesses introduced in the main paper to classify the corresponding causal maps. We will take the basis $|H\rangle$, $|V\rangle$ of eigenstates of the Pauli operator σ_z (which corresponds to horizontal and vertical polarization states) as the basis defining the Choi isomorphism; that is,

$$\left|\Phi^+\right\rangle \equiv \frac{1}{\sqrt{2}}(|HH\rangle + |VV\rangle). \tag{10}$$

Example of Coh

We begin with the circuit in Fig. (d), which realizes an example of Coh. The circuit applies the partial swap gate from equation 2,

$$\mathcal{E}_{BF|DE}(\cdot) = U_{BF|DE}\ (\cdot)\ U^\dagger_{BF|DE}, \tag{11}$$

where

$$U_{BF|DE} = \tfrac{1}{\sqrt{2}}\mathbb{1}_{B|D} \otimes \mathbb{1}_{F|E} + \tfrac{i}{\sqrt{2}}\mathbb{1}_{B|E} \otimes \mathbb{1}_{F|D}. \tag{12}$$

The first term of supplementary equation 12 corresponds to the identity operation, which maps D to B and E to F, and the second term corresponds to the swap operation, which maps D to F and E to B.

Inserting supplementary equation 11 and supplementary equation 12 as well as equation 1 from the main text into supplementary equation 8, one can find an explicit expression for the causal map realized by the partial swap, $\mathcal{E}^{coh}_{CB|D}$. However, in order to derive the compact expression quoted in the main text (equation 3), we will consider a Kraus representation of the map: a set of operators W_k such that

$$\mathcal{E}^{coh}_{CB|D}(\cdot) = \sum_k W_k(\cdot)W_k^\dagger. \tag{13}$$

One possible choice of Kraus operators is given in terms of the unitary that defines $\mathcal{E}_{BF|DE}$ by

$$W_k = \langle k_F|\, U_{BF|DE}\left|\Phi^+\right\rangle_{CE}, \tag{14}$$

where $\{|k\rangle\}$ is an arbitrary orthonormal basis of the Hilbert space of F.

We take $k \in \{H, V\}$, ranging over eigenvectors of σ_z, which is the same basis that puts the initial state of CE in a simple form. Substituting supplementary equation 12, we obtain

$$\begin{aligned}W_k &= \sum_{m\in\{H,V\}} \langle k_F|\, U_{BF|DE}\, |mm\rangle_{CE}\\ &= \sum_{m\in\{H,V\}} \tfrac{1}{\sqrt{2}}\langle k_F|\, \mathbb{1}_{B|D} \otimes \mathbb{1}_{F|E}\, |mm\rangle_{CE}\\ &\quad + \tfrac{i}{\sqrt{2}}\langle k_F|\, \mathbb{1}_{B|E} \otimes \mathbb{1}_{F|D}\, |mm\rangle_{CE}\\ &= \frac{1}{\sqrt{2}}(A_k + iB_k),\end{aligned} \tag{15}$$

where we introduce the components

$$
\begin{aligned}
A_k &\equiv \sum_{m\in\{H,V\}} \tfrac{1}{\sqrt{2}} \langle k_F| \mathbb{1}_{B|D} \otimes \mathbb{1}_{F|E} |mm\rangle_{CE} \\
&= \tfrac{1}{\sqrt{2}} \mathbb{1}_{B|D} \otimes |k\rangle_C \qquad (16)\\
B_k &\equiv \sum_{m\in\{H,V\}} \tfrac{1}{\sqrt{2}} \langle k_F| \mathbb{1}_{B|E} \otimes \mathbb{1}_{F|D} |mm\rangle_{CE} \\
&= |\Phi^+\rangle_{CB} \langle k|_D \,. \qquad (17)
\end{aligned}
$$

Note that the A_k contain only the two-qubit identity operator, which we expect to implement a purely cause-effect relation (the first term in supplementary equation 12), whereas the B_k contain only the swap operator, which we expect to realize a purely common-cause relation (the second term in supplementary equation 12).

In terms of the A_k and B_k, we have

$$
\begin{aligned}
\mathcal{E}^{coh}_{CB|D}(\cdot) &= \sum_k \frac{1}{2}(A_k + iB_k)(\cdot)(A_k + iB_k)^\dagger \qquad (18)\\
&= \sum_k \frac{1}{2} A_k(\cdot)A_k^\dagger + \frac{1}{2} B_k(\cdot)B_k^\dagger \\
&\quad - \frac{i}{2}\left\{ A_k(\cdot)B_k^\dagger - B_k(\cdot)A_k^\dagger \right\}.
\end{aligned}
$$

The effect of the first term is

$$
\begin{aligned}
\sum_k A_k(\cdot)A_k^\dagger &= \tfrac{1}{2}\sum_k |k\rangle\langle k|_C \otimes \mathbb{1}_{B|D}(\cdot)\mathbb{1}^\dagger_{B|D} \\
&= \tfrac{1}{2}\mathbb{1}_C \otimes \mathcal{I}_{B|D}(\cdot) \equiv \mathcal{E}^{ce}_{CB|D}(\cdot), \qquad (19)
\end{aligned}
$$

that is, it applies the identity channel from D to B, which is our example of a purely cause-effect relation. The effect of the second term is

$$
\begin{aligned}
\sum_k B_k(\cdot)B_k^\dagger &= |\Phi^+\rangle\langle\Phi^+|_{CB} \otimes \sum_k \langle k_D|(\cdot)|k_D\rangle \\
&= |\Phi^+\rangle\langle\Phi^+|_{CB}\, \mathrm{Tr}_D(\cdot) \equiv \mathcal{E}^{cc}_{CB|D}(\cdot), \qquad (20)
\end{aligned}
$$

that is, it traces out D and prepares the state $|\Phi^+\rangle$ on CB, which is our example of a purely common-cause relation. Finally, the cross terms take the form

$$
\begin{aligned}
&-\frac{i}{2}\sum_k \left\{ A_k(\cdot)B_k^\dagger - B_k(\cdot)A_k^\dagger \right\} \qquad (21)\\
&= -\frac{i}{2}\sum_k \Big\{ \left(\tfrac{1}{\sqrt{2}}|k\rangle_C \otimes \mathbb{1}_{B|D}\right)(\cdot)\left(\langle\Phi^+|_{CB} \otimes |k\rangle_D\right) \\
&\quad - \left(|\Phi^+\rangle_{CB} \otimes \langle k|_D\right)(\cdot)\left(\tfrac{1}{\sqrt{2}}\langle k|_C \otimes \mathbb{1}_{B|D}\right) \Big\} \\
&= -\, i\left[\tfrac{1}{2}\mathbb{1}_C \otimes \mathcal{I}_{B|D}(\cdot)\right] |\Phi^+\rangle\langle\Phi^+|_{CB} \\
&\quad + i\, |\Phi^+\rangle\langle\Phi^+|_{CB} \left[\tfrac{1}{2}\mathbb{1}_C \otimes \mathcal{I}_{B|D}(\cdot)\right] \Big\},
\end{aligned}
$$

which completes the derivation of equation 3 in the main paper.

The Choi representation of this causal map is then calculated using supplementary equation 9,

$$
\begin{aligned}
\tau^{coh}_{CBD} &= \frac{1}{2}\left(\tfrac{1}{2}\mathbb{1}_C \otimes |\Phi^+\rangle\langle\Phi^+|_{BD}\right) + \frac{1}{2}\left(|\Phi^+\rangle\langle\Phi^+|_{CB} \otimes \tfrac{1}{2}\mathbb{1}_D\right) \\
&\quad - \frac{i}{2}\Big\{ \left(\mathbb{1}_C \otimes |\Phi^+\rangle\langle\Phi^+|_{BD}\right)\cdot\left(|\Phi^+\rangle\langle\Phi^+|_{CB} \otimes \mathbb{1}_D\right) \qquad (22)\\
&\quad - \left(|\Phi^+\rangle\langle\Phi^+|_{CB} \otimes \mathbb{1}_D\right)\cdot\left(\mathbb{1}_C \otimes |\Phi^+\rangle\langle\Phi^+|_{BD}\right) \Big\}.
\end{aligned}
$$

The Choi state in supplementary equation 22 contains all the information required to characterize the causal structure. In order to evaluate our witnesses, we will calculate the induced states associated with finding certain states on C, B or D. Letting Π^b denote the projector associated with an eigenvalue b in a particular measurement on system B, we use

$$\tau^b_{CD} = \frac{1}{\mathrm{Tr}\left[\Pi^b_B \cdot \tau^{coh}_{CBD}\right]} \mathrm{Tr}_B\left[\Pi^b_B \cdot \tau^{coh}_{CBD}\right], \tag{23}$$

and similarly for a projector Π^c on C,

$$\tau^c_{BD} = \frac{1}{\mathrm{Tr}\left[\Pi^c_C \cdot \tau^{coh}_{CBD}\right]} \mathrm{Tr}_C\left[\Pi^c_C \cdot \tau^{coh}_{CBD}\right]. \tag{24}$$

The expression for the induced state on CB generated by an input Π^d on D differs from the above by a partial transpose, and the renormalization factor to ensure unit trace is always d_D:

$$\tau^d_{CB} = \mathcal{E}^{coh}_{CB|D}\left(\Pi^d_D\right) = d_D \mathrm{Tr}_D\left[\tau^{coh}_{CBD} T_D\left(\Pi^d_D\right)\right]. \tag{25}$$

If one prepares D in the state $|H\rangle$, then the state on CB is $\tau^H_{CB} = \frac{3}{4}|\psi\rangle\langle\psi| + \frac{1}{4}|VH\rangle\langle VH|$ where $|\psi\rangle = \frac{2}{\sqrt{6}}|HH\rangle + e^{i\pi/4}\frac{1}{\sqrt{3}}|VV\rangle$, and one can see that τ^H_{CB} is entangled. The same holds if one prepares $|V\rangle$ on D instead. The causal map $\mathcal{E}^{coh}_{CB|D}$ is therefore quantum in the cause-effect pathway. If one measures C and selects for the state $|H\rangle$, the resulting map from D to B is Choi-isomorphic (up to normalization) to the state $\tau^H_{BD} = \frac{3}{4}\left|\tilde{\psi}\right\rangle\left\langle\tilde{\psi}\right| + \frac{1}{4}|HV\rangle\langle HV|$, where $\left|\tilde{\psi}\right\rangle = \frac{2}{\sqrt{6}}|HH\rangle + e^{-i\pi/4}\frac{1}{\sqrt{3}}|VV\rangle$. One can see that τ^H_{BD} is entangled. The same result is found if the measurement on C finds $|V\rangle$, and therefore the causal map is quantum in the common-cause pathway.

Finding B in the state $|H\rangle$ also induces entanglement between C and D, as was already shown in the main text. For completeness, we note that, if one finds $|V\rangle$ instead, the induced Choi state on CD is

$$\tau^V_{CD} = \frac{1}{2}|VV\rangle\langle VV| + \frac{1}{2}|\tilde{\varphi}\rangle\langle\tilde{\varphi}|, \tag{26}$$

with $|\tilde{\varphi}\rangle \equiv \frac{1}{\sqrt{2}}(|HV\rangle + i|VH\rangle)$, which is also entangled. As a result, the causal map $\mathcal{E}^{coh}_{CB|D}$ satisfies all the requirements for the class COH: it is quantum in both the cause-effect and the common-cause pathways and, furthermore, exhibits a quantum Berkson effect.

Example of ProbQ

The circuit realizing an example of the class ProbQ is presented Fig. (c). This circuit implements a probabilistic mixture of identity and swap,

$$\begin{aligned}\mathcal{E}_{BF|DE}(\cdot) = &\frac{1}{2}(\mathbb{1}_{B|D}\otimes\mathbb{1}_{F|E})\,(\cdot)\,\left(\mathbb{1}_{B|D}\otimes\mathbb{1}_{F|E}\right)\\ &+\frac{1}{2}(\mathbb{1}_{B|E}\otimes\mathbb{1}_{F|D})\,(\cdot)\,\left(\mathbb{1}_{B|E}\otimes\mathbb{1}_{F|D}\right).\end{aligned} \tag{27}$$

Using supplementary equation 8, we find

$$\mathcal{E}_{CB|D}(\cdot) = \frac{1}{2}\mathcal{E}^{ce}_{CB|D}(\cdot) + \frac{1}{2}\mathcal{E}^{cc}_{CB|D}(\cdot), \tag{28}$$

where $\mathcal{E}^{ce}_{CB|D}$ and $\mathcal{E}^{cc}_{CB|D}$ are defined in supplementary equation 19 and supplementary equation 20 respectively. This shows explicitly that the map is a probabilistic mixture of a purely cause-effect term and a purely common-cause term.

By supplementary equation 9, the Choi state is

$$\tau_{CBD} = \frac{1}{2}\frac{1}{2}\mathbb{1}_C\otimes\left|\Phi^+\right\rangle\left\langle\Phi^+\right|_{BD} + \frac{1}{2}\left|\Phi^+\right\rangle\left\langle\Phi^+\right|_{CB}\otimes\frac{1}{2}\mathbb{1}_D. \tag{29}$$

Finding C in the state $|H\rangle$ implies $\tau^H_{BD} = \frac{1}{2}|\Phi^+\rangle\langle\Phi^+|_{BD} + \frac{1}{2}|H\rangle\langle H|_B\otimes\frac{1}{2}\mathbb{1}_D$, which is entangled, and similarly if C is found in the state $|V\rangle$. The causal map is therefore quantum in the cause-effect pathway. If we prepare $|H\rangle$ on D, then the state on CB is $\tau^H_{CB} = \frac{1}{2}|\Phi^+\rangle\langle\Phi^+|_{CB} + \frac{1}{2}\left(\frac{1}{2}\mathbb{1}_C\otimes|H\rangle\langle H|_B\right)$, which is also entangled. The same holds when preparing $|V\rangle$ on D, and consequently the causal map is quantum in the common-cause pathway. It follows that the causal map is in the class ProbQ.

Causal maps that are classical on both pathways

A general way to realize causal maps that are classical on both the cause-effect and common-cause pathway is to insert completely dephasing channels before and after the gate $\mathcal{E}_{BF|DE}$. The generic completely dephasing channel takes the form

$$\Delta_{\hat{n}}(\rho) = \frac{1}{2}\rho + \frac{1}{2}\left([\hat{n}\cdot\vec{\sigma}]\rho[\hat{n}\cdot\vec{\sigma}]\right), \tag{30}$$

where $\vec{\sigma}$ is the vector of Pauli observables and the Bloch vector $\hat{n}$ specifies the eigenbasis on which we dephase. The dephasing effectively reduces the qubits B, D, E, and F to classical binary variables, which we denote b, d, e, f, and reduces the map $\mathcal{E}_{BF|DE}$ to a conditional probability distribution $P(bf|de)$: letting $|b\rangle$ denote the elements of a preferred basis of $\mathcal{H}_B$ – namely, the eigenbasis of $\hat{n}_B \cdot \vec{\sigma}$ –, and similarly for F, D and E, we can write

$$\begin{aligned}\mathcal{E}_{BF|DE}(\rho_{DE}) = \sum_{b,d,e,f} & P(bf|de)\,|b\rangle\langle b| \otimes |f\rangle\langle f| \\ & \times \mathrm{Tr}_{DE}\left(|d\rangle\langle d| \otimes |e\rangle\langle e|\,\rho_{DE}\right).\end{aligned} \tag{31}$$

The dephasing on E also effectively reduces C to a classical binary variable, since C is only related to other variables in the problem via E. We denote this variable by c and the corresponding preferred basis (which generally depends on the initial joint state ρ_{CE}) by $|c\rangle$. Substituting supplementary equation 31 into supplementary equation 8, we find that the causal map takes the form

$$\mathcal{E}_{CB|D}(\rho_D) \equiv \sum_{c,b,d} P(cb|d)\,|c\rangle\langle c| \otimes |b\rangle\langle b| \times \mathrm{Tr}_D\left(|d\rangle\langle d|\,\rho_D\right). \tag{32}$$

Note that, since the prescription for deriving the causal map $\mathcal{E}_{CB|D}$ from $\mathcal{E}_{BF|DE}$ and ρ_{CE} involves tracing out system F, we find this form of the causal map independently of whether we actually apply dephasing on F: dephasing on D, E and B is sufficient.

It follows that the corresponding Choi state takes the form

$$\tau_{CBD} = \sum_{c,b,d} P(cb|d)u(d)\,|c\rangle\langle c| \otimes |b\rangle\langle b| \otimes |\tilde{d}\rangle\langle\tilde{d}|, \tag{33}$$

where $u(d)$ denotes the uniform distribution over d and $|\tilde{d}\rangle$ is related to $|d\rangle$ by complex conjugation in the basis that defines the Choi isomorphism (in our case, $\{|H\rangle, |V\rangle\}$). Similarly, the induced states, for a preparation $\rho_D = |d\rangle\langle d|$, projection $\Pi^c_C = |c\rangle\langle c|$, and projection $\Pi^b_B = |b\rangle\langle b|$ are given by the operators

$$\begin{aligned}\tau^d_{CB} &= \sum_{c,b} P^d(c,b)\,|c\rangle\langle c| \otimes |b\rangle\langle b| \\ \tau^c_{BD} &= \sum_{b,d} P^c(b,d)\,|b\rangle\langle b| \otimes |\tilde{d}\rangle\langle\tilde{d}|, \\ \tau^b_{CD} &= \sum_{c,d} P^b(c,d)\,|c\rangle\langle c| \otimes |\tilde{d}\rangle\langle\tilde{d}|.\end{aligned} \tag{34}$$

with

$$\begin{aligned}P^d(c,b) &= P(cb|d) \\ P^c(b,d) &= P(cb|d)u(d)/\left[\sum_{bd} P(cb|d)u(d)\right] \\ P^b(c,d) &= P(cb|d)u(d)/\left[\sum_{cd} P(cb|d)u(d)\right].\end{aligned} \tag{35}$$

Any operator of the form of supplementary equation 34 is separable. Therefore, by our criterion, such dephased causal maps are not quantum in either pathway.

Example of PROBC

A circuit realizing an example of PROBC is presented in Fig. (a). It applies complete dephasing channels to D and E only. However, note that B and F are obtained from D and E by either the two-qubit identity channel or the swap. This implies that B and F are also effectively classical, on the same bases on which we dephase D and E. The gate therefore can be expressed in the form of supplementary equation 31, with

$$P(bf|de) = \frac{1}{2}\delta_{b,d}\delta_{f,e} + \frac{1}{2}\delta_{b,e}\delta_{f,d}, \tag{36}$$

where $\delta_{x,y}$ denotes the Kronecker delta function over variables x, y. The variables d and e are mapped either to b and f or to f and b, respectively, with equal probability: a probabilistic mixture of classical identity and swap. The causal map is therefore effectively described by a classical probability distribution, as in supplementary equation 32, with

$$P(cb|d) = \frac{1}{2}u(c)\delta_{b,d} + \frac{1}{2}\delta_{c,b}u(c), \tag{37}$$

where $u(x)$ denotes the uniform distribution of the variable x. The dephasing ensures that the common-cause and cause-effect components of the causal map are classical, and the form of $\mathcal{E}_{CB|D}$ makes it clear that this is a probabilistic mixture of classical cause-effect and common-cause relations. The causal map is therefore in the class PROBC.

Example of PHYSC

A circuit realizing an example of PHYSC is presented in Fig. (b). Again, we explicitly apply dephasing channels only to D and E, but note that the classical XNOR gate, which generates B in the left-hand panel, implicitly defines a preferred basis – in other words: if B is the output of a classical XNOR (Not-XOR: $b = -de$ for $d, e \in \{-1, 1\}$), then B must be (effectively) classical. In the right-hand panel, B is prepared in the maximally mixed state, which can also be described as effectively classical. The same holds for F, and we can therefore again express the causal map in terms of a classical conditional distribution,

$$P(bf|de) = \frac{1}{2}\delta_{b,-de}u(f) + \frac{1}{2}u(b)\delta_{f,-de} \ , \tag{38}$$

The gate either sets $b = -de$ and generates f at random or vice versa. This leads to a causal map of the form of supplementary equation 32, with

$$P(cb|d) = \frac{1}{2}u(c)u(b) + \frac{1}{2}u(c)\delta_{b,-cd}. \tag{39}$$

Even though b is completely unaffected by d and e in the first term, in the second term b depends nontrivially on both inputs. One can see that this makes the causal map a physical mixture: indeed, the induced state τ^b_{CD} in this case is given by supplementary equation 34 with

$$P^b(cd) = \frac{1}{2}u(c)u(d) + \frac{1}{2}u(c)\delta_{b,-cd}. \tag{40}$$

The mutual information between c and d in this distribution is 0.19 bits for either value of b. By contrast, we will show in a later section that the induced mutual information between binary variables c and d for any probabilistic mixture of common-cause and cause-effect with uniform prior distributions (which is the case here) is at most 0.12 bits. It follows that the present example must be a physical mixture, and noting furthermore that the causal map is classical in both pathways, we conclude that it belongs to the class PHYSC.

Proof that COH *is a strict subset of* PHYSQ

Based on the previous scenarios, one can now see that COH is in fact a strict subset of PHYSQ. To show this, we will explicitly construct a causal map that belongs to PHYSQ but not to COH. To wit, consider a probabilistic mixture of

our examples of ProbQ and PhysC, with a small weight ϵ for the latter:

$$\begin{aligned}\mathcal{E}_{CB|D}(\cdot) = \frac{1-\epsilon}{2}\Big(\tfrac{1}{2}\mathbb{1}_C \otimes \mathcal{I}_{B|D}(\cdot) + \left|\Phi^+\right\rangle\left\langle\Phi^+\right|_{CB} \times \mathrm{Tr}_D(\cdot)\Big) \\ + \frac{\epsilon}{2}\Big(\tfrac{1}{2}\mathbb{1}_C \otimes \tfrac{1}{2}\mathbb{1}_B \times \mathrm{Tr}_D(\cdot) \\ + \sum_{c,b,d} u(c)\delta_{b,-cd}\left|c\right\rangle\left\langle c\right| \otimes \left|b\right\rangle\left\langle b\right| \times \mathrm{Tr}_D\left(\left|d\right\rangle\left\langle d\right| \ \cdot\right)\Big),\end{aligned} \tag{41}$$

where we take the preferred bases for the example of PhysC, $|c\rangle$, $|b\rangle$ and $|d\rangle$, to each be the eigenbasis of σ_z.

In this case, the resulting causal map is still quantum on both pathways: indeed, finding C in the state $|H\rangle$ implies

$$\begin{aligned}\tau^H_{BD} = \frac{1-\epsilon}{2}\left|\Phi^+\right\rangle\left\langle\Phi^+\right|_{BD} + \frac{1-\epsilon}{2}\left|H\right\rangle\left\langle H\right|_B \otimes \tfrac{1}{2}\mathbb{1}_D \\ + \frac{\epsilon}{2}\tfrac{1}{2}\mathbb{1}_B \otimes \tfrac{1}{2}\mathbb{1}_D + \frac{\epsilon}{2}\sum_{b,d} u(d)\delta_{b,-d}\left|b\right\rangle\left\langle b\right| \otimes |\tilde{d}\rangle\langle\tilde{d}|,\end{aligned} \tag{42}$$

which is entangled for a range of ϵ, and similarly for finding C in the state $|V\rangle$. The causal map is therefore quantum in the cause-effect pathway. If we prepare $|H\rangle$ on D, then the state on CB is

$$\begin{aligned}\tau^H_{CB} = \frac{1-\epsilon}{2}\left|\Phi^+\right\rangle\left\langle\Phi^+\right|_{CB} + \frac{1-\epsilon}{2}\tfrac{1}{2}\mathbb{1}_C \otimes \left|H\right\rangle\left\langle H\right|_B \\ + \frac{\epsilon}{2}\tfrac{1}{2}\mathbb{1}_C \otimes \tfrac{1}{2}\mathbb{1}_B + \frac{\epsilon}{2}\sum_{c,b} u(c)\delta_{b,-c}\left|c\right\rangle\left\langle c\right| \otimes \left|b\right\rangle\left\langle b\right|,\end{aligned} \tag{43}$$

which is also entangled. The same holds for preparing $|V\rangle$ on D, and consequently the causal map is quantum in the common-cause pathway. However, the causal map cannot be realized by a probabilistic mixture of purely common-cause and purely cause-effect relations, since the witness of physical mixture is $\epsilon/4$, i.e. non-zero for all valid values of ϵ. (The fact that the last term, $b = -cd$, has b depending simultaneously on c and d is also suggestive of a physical mixture, but not conclusive.) Consequently, the map belongs to PhysQ.

On the other hand, no measurement outcome on B implies entanglement on CD: since every term in the expression for $\mathcal{E}_{CB|D}$ has the form of a tensor product between C and D, the state induced when selecting for any state on B must be separable. It follows that the map is not in Coh.

Supplementary Note 3: Implementing the examples using a single experimental set-up

In the following section, we show how the single set-up in Fig. can experimentally realize our examples of the classes Coh (hence PhysQ), ProbQ, ProbC, and PhysC. We first describe the implementation of the partial swap gate, which allows us to realize the example of Coh from Fig. (d). Next we describe how this gate can be modified in order to realize the example of ProbQ from Fig. (c). We then move on to discuss how applying complete dephasing channels on D, E and B yields causal maps where both pathways are classical. We show that by modifying the type of dephasing, we can realize the examples of ProbC and PhysC (up to a sign change) presented in Fig. (a,b).

Example of Coh

We begin by describing how the partial swap gate is implemented experimentally. In the set-up of Fig. , two photons are input at D and E and measured in coincidence at B and F. If the photons input at D and E are indistinguishable, then when they arrive at the first beam splitter, they will bunch if their polarization state lies in the symmetric subspace (spanned by the triplet basis), while they will anti-bunch if their polarization state lies in the anti-symmetric subspace (singlet). If they bunch, then a coincidence at B and F can only be obtained if both photons take the clockwise path in the Sagnac interferometer. However, if they anti-bunch, the two photons will take opposite paths. The photon travelling along counterclockwise path will acquire an extra phase, denoted θ, due to the glass windows, while the other, on the clockwise path, acquires no extra phase.

In this configuration, the gate applies a phase difference between the symmetric and anti-symmetric subspaces of the photon state. Recall that the projectors onto these subspaces can be written as linear combinations of the identity

and swap operators,

$$\begin{aligned}\mathbb{S} &= \frac{1}{2}\left(\mathbb{1}_{B|D}\otimes\mathbb{1}_{F|E}+\mathbb{1}_{B|E}\otimes\mathbb{1}_{F|D}\right)\\ \mathbb{A} &= \frac{1}{2}\left(\mathbb{1}_{B|D}\otimes\mathbb{1}_{F|E}-\mathbb{1}_{B|E}\otimes\mathbb{1}_{F|D}\right),\end{aligned} \tag{44}$$

so that the gate takes the form

$$\mathcal{E}_{BF|DE}(\rho) = \left(\mathbb{S}+e^{i\theta}\mathbb{A}\right)\rho\left(\mathbb{S}+e^{i\theta}\mathbb{A}\right)^{\dagger}. \tag{45}$$

Substituting the expressions for $\mathbb{S}$ and $\mathbb{A}$, we find

$$\begin{aligned}\mathbb{S}+&e^{i\theta}\mathbb{A} = \\ =&\frac{\mathbb{1}_{B|D}\otimes\mathbb{1}_{F|E}+\mathbb{1}_{B|E}\otimes\mathbb{1}_{F|D}}{2}\\ &+e^{i\theta}\frac{\mathbb{1}_{B|D}\otimes\mathbb{1}_{F|E}-\mathbb{1}_{B|E}\otimes\mathbb{1}_{F|D}}{2}\\ =&e^{i\theta/2}\left(\cos(\theta/2)\mathbb{1}_{B|D}\otimes\mathbb{1}_{F|E}-i\sin(\theta/2)\mathbb{1}_{B|E}\otimes\mathbb{1}_{F|D}\right)\end{aligned} \tag{46}$$

Thus, by adjusting the phase of the Sagnac interferometer to $\theta=-\pi/2$, we obtain a gate $\mathcal{E}_{BF|DE}$ that implements the partial swap unitary $U_{BF|DE}$ given by equation 2 in the main text. This allows us to build the circuit in Fig. (d) and realize our example of the class Coh.

Example of ProbQ

In order to realize our example of the class ProbQ, we modify the experimental set-up as follows. If we delay photon E with respect to photon D, which can be accomplished using a translation stage, then the two-photon interference at the beam splitter no longer occurs. The two pathways that lead to a coincidence measurement at B and F remain the same, but they no longer act coherently. The gate can instead be understood to project into the symmetric and anti-symmetric subspaces: in terms of the operators $\mathbb{S}$ and $\mathbb{A}$ defined in supplementary equation 44,

$$\mathcal{E}_{BF|DE}(\cdot) = \mathbb{S}(\cdot)\mathbb{S}+\mathbb{A}(\cdot)\mathbb{A}. \tag{47}$$

Substituting the expressions from supplementary equation 44, we find that this expression is equivalent to the probabilistic mixture of identity and swap of supplementary equation 27:

$$\begin{aligned}\mathcal{E}_{BF|DE}(\cdot) = &\frac{1}{2}(\mathbb{1}_{B|D}\otimes\mathbb{1}_{F|E})\,(\cdot)\,\left(\mathbb{1}_{B|D}\otimes\mathbb{1}_{F|E}\right)\\ &+\frac{1}{2}(\mathbb{1}_{B|E}\otimes\mathbb{1}_{F|D})\,(\cdot)\,\left(\mathbb{1}_{B|E}\otimes\mathbb{1}_{F|D}\right).\end{aligned} \tag{48}$$

We note that, unlike the previous case, the overall gate does not depend on the relative phase θ of the clockwise and anti-clockwise paths through the Sagnac interferometer. Here, the beam splitter reflectivity adjusts the relative weights of identity and swap. For a 50-50 beam splitter, as used in the experiment, both the identity and swap operations will have equal weights and we obtain the circuit of Fig. (c), which implements an example of the class ProbQ.

Example of ProbC

In order to experimentally realize our example of the class ProbC, we modify the gate $\mathcal{E}_{BF|DE}$ from supplementary equation 45 by applying complete dephasing along the $\hat{z}$ axis to E, D, and B, so that

$$\begin{aligned}&\mathcal{E}_{BF|DE}(\cdot) = \\ &(\Delta^B_{\hat{z}}\otimes\mathcal{I}_F)\left(U_{BF|DE}\left((\Delta^D_{\hat{z}}\otimes\Delta^E_{\hat{z}})(\cdot)\right)U^{\dagger}_{BF|DE}\right).\end{aligned} \tag{49}$$

Using supplementary equation 8, the causal map is found to be

$$\mathcal{E}_{CB|D}(\cdot) = \frac{1}{2}\tfrac{1}{2}\mathbb{1}_C \otimes \Delta^B_{\hat{z}} \circ \mathcal{I}_{B|D}(\cdot) \\ + \frac{1}{2}\left(\Delta^C_{\hat{z}} \otimes \Delta^B_{\hat{z}}\right)\left(|\Phi^+\rangle\langle\Phi^+|_{CB}\right)\mathrm{Tr}_D(\cdot)\,. \tag{50}$$

One can see that this causal map takes the effectively classical form of supplementary equation 32, with $|c\rangle$, $|b\rangle$ and $|d\rangle$ all denoting eigenstates of σ_z, and

$$P(cb|d) = \frac{1}{2}u(c)\delta_{b,d} + \frac{1}{2}\delta_{c,b}u(c), \tag{51}$$

which is exactly the same as in our example of Fig. (a). As we pointed out in the previous discussion of this example (around supplementary equation 37), the causal map is classical in both pathways (due to the dephasing) and manifestly takes the form of a probabilistic mixture, hence it belongs to ProbC.

Example of PhysC

The class PhysC is experimentally realized by applying complete dephasing in the eigenbases of σ_x on E, σ_y on D and σ_z on B. This choice of bases ensures that the witness of physical mixture, which is evaluated using only measurements of these particular observables, remains unchanged by the dephasing. It therefore ensures that we continue to realize a physical mixture while eliminating the coherence in the cause-effect and common-cause paths. Combining the dephasing channels with the partial swap unitary, we can write the overall two-qubit gate in this scenario as

$$\mathcal{E}_{BF|DE}(\cdot) = \\ (\Delta^B_{\hat{z}} \otimes \mathcal{I}_F)\left(U_{BF|DE}\left((\Delta^D_{\hat{y}} \otimes \Delta^E_{\hat{x}})(\cdot)\right)U^\dagger_{BF|DE}\right). \tag{52}$$

Since the dephasing introduces a different preferred basis for each of the qubits, we will use the notation $|c_x\rangle$, $|d_y\rangle$ and $|b_z\rangle$, with $\{c, d, b\} \in \pm 1$, for the eigenstates of the Pauli operators σ_x, σ_y and σ_z, respectively. Inserting $\mathcal{E}_{BF|DE}$ into supplementary equation 8, one can obtain the causal map $\mathcal{E}_{CB|D}$, which takes the special form shown in supplementary equation 32 with $P(cb|d)$ given by supplementary equation 39. Similarly, the corresponding Choi state is diagonal in the bases $|\pm_x\rangle$ on C, $|\pm_z\rangle$ on B and $|\pm_y\rangle$ on D:

$$\tau_{CBD} = \tfrac{1}{16}\mathbb{1}_{CBD} \\ + \tfrac{1}{8}|+_x\rangle\langle+_x|_C \otimes |-_z\rangle\langle-_z|_B \otimes |-_y\rangle\langle-_y|_D \\ + \tfrac{1}{8}|-_x\rangle\langle-_x|_C \otimes |-_z\rangle\langle-_z|_B \otimes |+_y\rangle\langle+_y|_D \\ + \tfrac{1}{8}|+_x\rangle\langle+_x|_C \otimes |+_z\rangle\langle+_z|_B \otimes |+_y\rangle\langle+_y|_D \\ + \tfrac{1}{8}|-_x\rangle\langle-_x|_C \otimes |+_z\rangle\langle+_z|_B \otimes |-_y\rangle\langle-_y|_D \tag{53}$$

(As pointed out after supplementary equation 33, the basis $|\tilde{d}\rangle$ that diagonalizes the Choi state is related to the basis $|d\rangle$ that diagonalizes the causal map by complex conjugation in the basis that defines the Choi isomorphism. In our case, $|d\rangle$ are eigenstates of σ_y, and the Choi isomorphism is defined by the eigenbasis of σ_z, hence $|\tilde{d}\rangle$ are also eigenstates of σ_y, albeit with the opposite eigenvalues.)

Since the output B depends on whether C and D are correlated or anti-correlated, the causal structure cannot be described by a probabilistic mixture of purely cause-effect and purely common-cause mechanisms. Indeed, one can see that the causal map is effectively classical, since it takes the form of supplementary equation 32, and the classical conditional distribution $P(cb|d)$ has the same form as supplementary equation 39 (up to an exchange of positive and negative correlations). Since both pathways of the causal map are classical, we conclude that it belongs to PhysC.

Supplementary Note 4: Reconstructing the causal map and obtaining the negativity from experimental data

This section details how we reconstruct causal maps from experimental data using a maximum likelihood estimation. The analysis is based on [4].

The measurement statistics obtained in the experiment take the form of count numbers for different combinations of wave plate orientations. We measure the Pauli observable σ_s on C and σ_u on B, denoting the resulting eigenvalues by c and b, respectively, and prepare the d eigenstate of σ_t on D, where $s, t, u \in \{1, 2, 3\}$ range over $\sigma_1 \equiv \sigma_x$, $\sigma_2 \equiv \sigma_y$ and $\sigma_3 \equiv \sigma_z$. Since the orientation of the wave plates encodes both the choice of observable and the selected eigenstate, the outcome in this case is not one of two possible eigenvalues, but rather whether the photon reaches the detector in the end, indicating that it was in the desired eigenstate. The observed count numbers for the wave plate orientations specified by $cbdstu$ are denoted $\tilde{P}^{\mathrm{obs}}(cbdstu)$. The expected count numbers for wave plate orientations encoding s, c, t, d and u, b are therefore proportional to the joint probabilities of realizing the eigenvalues c, b, d and the choices of Pauli operators s, t, u. We denote the expected count numbers predicted by the fitting model by

$$\tilde{P}^{\mathrm{fit}}(cbdstu) = N P^{\mathrm{fit}}(cbdstu), \tag{54}$$

where $P^{\mathrm{fit}}(cbdstu)$ is the joint probability distribution predicted by the fitting model and N is the number of runs of the experiment.

Let us now relate the joint probability distribution $P^{\mathrm{fit}}(cbdstu)$ to the model parameters, in particular the Choi state τ_{CBD} which represents the causal map. To this end, we introduce the notation $\Pi^{s,c}$ for the projector onto the $c \in \{\pm 1\}$ eigenstate of the Pauli operator σ_s, with $s \in \{1, 2, 3\}$. The conditional probability of finding eigenvalues c, b, given that one chose Pauli observables s, t, u and prepared the d eigenstate on D, can then be written in terms of the causal map and its Choi state as

$$\begin{aligned} P^{\mathrm{fit}}(cb|dstu) &\equiv \mathrm{Tr}\left[\Pi_C^{s,c} \otimes \Pi_B^{u,b} \mathcal{E}_{CB|D}\left(\Pi_D^{t,d}\right)\right] \\ &\equiv 2\mathrm{Tr}\left[T_D\left(\tau_{CBD}\right) \Pi_C^{s,c} \otimes \Pi_B^{u,b} \otimes \Pi_D^{t,d}\right], \end{aligned} \tag{55}$$

where T_D denotes the transpose with respect to the input system D. In our experiment, we choose which eigenstate d to prepare by rotating the wave plates after the polarizing beam-splitter, with each setting being implemented for an equal period of time. Under the assumptions of a constant rate of photon production (on average) and equal transmission efficiency of the wave plates with different settings, this can be modelled by simply taking the probability of each eigenvalue to be $P(d|t) = \frac{1}{2}$ for $d = \pm 1$, $t = 1, 2, 3$. In this case, the probabilities of eigenvalues c, b, d given the settings (choices of eigenbasis) s, t, u become

$$\begin{aligned} P^{\mathrm{fit}}(cbd|stu) &= P^{\mathrm{fit}}(cb|dstu) P^{\mathrm{fit}}(d|t) \\ &= \mathrm{Tr}\left[\tau_{CBD} \cdot \Pi_C^{s,c} \otimes \Pi_B^{u,b} \otimes T_D\left(\Pi_D^{t,d}\right)\right], \end{aligned} \tag{56}$$

where we note that the probability of outcome d in the measurement on D is independent of the settings of the other two measurements, that is, $P(d|stu) = P(d|t)$. Finally, we note that the choice of observables s, t, u in our experiment is made at random, so that $P(stu) = \frac{1}{27}$ for all values of s, t, u. Using the chain rule $P(cbdstu) = P(cbd|stu)P(stu)$, we can finally write the expected count numbers in terms of the model parameters:

$$\tilde{P}^{\mathrm{fit}}(cbdstu) = \mathrm{Tr}\left[\frac{N}{27} \tau_{CBD} \cdot \Pi_C^{s,c} \otimes \Pi_B^{u,b} \otimes T_D\left(\Pi_D^{t,d}\right)\right]. \tag{57}$$

The operator τ_{CBD} that parametrizes the model is subject to certain consistency constraints: as a Choi state, it must be positive semi-definite and have trace one, while the combination $\frac{N}{27}\tau_{CBD}$ need only be positive, but not normalized. Following Ref. [5], this is achieved with the following parameterization:

$$\tau_{CBD} = \frac{N}{27} J_{CBD}^{\dagger} J_{CBD}, \tag{58}$$

where J_{CBD} is an 8x8 lower triangular matrix with real diagonal elements, specified by 64 real parameters. This form, known as the Cholesky decomposition, is positive-semidefinite by design, and, by varying over J_{CBD}, ranges over all positive operators. We normalize to trace one after the optimization by dividing by $\mathrm{Tr}(J_{CBD}^{\dagger} J_{CBD})$.

A second constraint arises due to the particular configuration of our experimental setup: since the preparation D occurs after the measurement of C, the input at D cannot have any causal influence on the measurement outcome at C. Therefore, the marginal $\tau_{CD} \equiv \mathrm{Tr_B}(\tau_{CBD})$ must be independent of D,

$$\tau_{CD} = \rho_C \otimes \frac{\mathbb{1}}{2}, \tag{59}$$

where $\rho_C = \mathrm{Tr_{BD}}(\tau_{CBD})$.

We include this additional constraint in the least-squares fit by adding penalty functions to the residue, so that the overall argument becomes

$$\begin{aligned} \chi^2 = &\sum_{cbdstu} \frac{\left[\tilde{P}^{\text{obs}}(cbdstu) - \tilde{P}^{\text{fit}}(cbdstu)\right]^2}{\tilde{P}^{\text{fit}}(cbdstu)} \\ &+ \lambda \sum_{ij} \left|(\tau_{CD} - \rho_C \otimes \frac{\mathbb{1}}{2})_{ij}\right|^2 . \end{aligned} \tag{60}$$

To enforce these constraints but not overshadow the principal function, the value of the Lagrange multiplier, λ was selected heuristically to be 10^7.

Obtaining the negativity from experimental data

We now describe how to obtain the negativity of the induced states τ^d_{CB}, τ^c_{BD}, and τ^b_{CD} from experimental data. Since the method is similar for all three, we will only illustrate it for the last case. In order to measure entanglement in τ^b_{CD}, we first reconstruct the state as a 2-qubit operator on C and D, using only count numbers from runs in which we found a particular eigenstate $\Pi^{u,b}$ on B, denoted $\tilde{P}^{\text{obs}}(cdst|bu)$. Following the model from the least-squares reconstruction of the full causal map, and still assuming that the preparations on D can be modelled with $P(d) = \frac{1}{2}$, the joint count numbers take the form of supplementary equation 57. We post-select on an outcome b, assuming the measurement basis u to be fixed. This gives rise to the conditional distribution

$$\begin{aligned} &\tilde{P}^{\text{fit}}(cdst|bu) = \\ &= \frac{N}{9}\text{Tr}_{CD}\left[\frac{1}{P(b|u)}\text{Tr}_B\left(\Pi^{u,b}_B \tau_{CBD}\right)\Pi^{s,c}_C \otimes T_D\left(\Pi^{t,d}_D\right)\right] \\ &= \frac{N}{9}\text{Tr}_{CD}\left[\tau^b_{CD}\ \ \Pi^{s,c}_C \otimes T_D\left(\Pi^{t,d}_D\right)\right]. \end{aligned} \tag{61}$$

Although a full specification of the state on B on which we post-select specifies both the eigenvalue b and the choice of observable u, since the latter is assumed fixed, we suppress it and write simply τ^b_{CD}. The model against which we compare the observed count numbers can therefore be written as

$$\tilde{P}^{\text{fit}}(cdst|b) = \text{Tr}_{CD}\left[\frac{N}{9}\tau^b_{CD}\Pi^{s,c}_C \otimes T_D\left(\Pi^{t,d}_D\right)\right]. \tag{62}$$

The reconstruction method is then essentially the same as for the full causal maps: we parametrize τ^b_{CD} as a 4x4 lower triangular matrix with 16 real parameters and minimize the residue

$$\chi^2 = \sum_{cdst} \frac{\left[\tilde{P}^{\text{obs}}(cdst) - \tilde{P}^{\text{fit}}(cdst)\right]^2}{\tilde{P}^{\text{fit}}(cdst)}. \tag{63}$$

Once the optimal parameters have been found, the negativity, $\mathcal{N}$, of the reconstructed state τ^b_{CD} is calculated using equation 6. Negativities for the induced states τ^d_{CB} and τ^c_{BD} are calculated in a similar way.

Supplementary Note 5: Witness of physical mixture

In this section, we define a family of functions of the experimental statistics that witness physical mixtures of common-cause and cause-effect mechanisms. In other words, we seek functions that are zero for all probabilistic mixtures and non-zero for at least some physical mixtures. For simplicity, we restrict ourselves to the case of qubits.

The witness is defined in terms of the statistics of measurements of a single Pauli observable on each C and B and preparations of eigenstates of a third Pauli observable on D. That is, the settings s, t, u are fixed, with each choice giving rise to a different witness from the same family, and we omit them in the following for brevity. When calculating the witness, we choose the eigenvalue d for the preparation of D from the uniform distribution, $P(d) = \frac{1}{2}$ for $d = \pm 1$, and hence the joint probability distribution $P(cdb)$ takes the same form as in supplementary equation 56.

Properties of probabilistic mixtures

We begin by noting several mathematical properties of probabilistic mixtures that will be useful in the subsequent derivations.

As already shown, the Choi state representing a causal map that is a probabilistic mixture of common-cause and cause-effect can always be expressed as a sum of only two terms,

$$\tau_{CBD}^{prob} = p\rho_{CB} \otimes \frac{1}{2}\mathbb{1}_D + (1-p)\rho_C \otimes \tau_{BD}. \tag{64}$$

The first term represents the common-cause scenario, wherein we prepare a bipartite state ρ_{CB} and trace out D; hence the marginal on D of the Choi state is the completely mixed state. The state ρ_{CB} is obtained from the initial state ρ_{CE} by a CPTP map that takes E to B, hence the marginal on C is unchanged: $\mathrm{Tr}_B\rho_{CB} = \mathrm{Tr}_E\rho_{CE}$. The second term corresponds to a cause-effect scenario, in which case the marginal state on C is simply the marginal of the initial bipartite state ρ_{CE}, $\rho_C = \mathrm{Tr}_E\rho_{CE}$. Meanwhile, τ_{BD} is the Choi state corresponding to a CPTP map from D to B, hence its marginal on D is again the completely mixed state. In summary, the marginals of the two terms on C and D, respectively, are equal:

$$\mathrm{Tr}_B\rho_{CB} = \mathrm{Tr}_E\rho_{CE} = \rho_C, \tag{65}$$

$$\mathrm{Tr}_B\tau_{BD} = \frac{1}{2}\mathbb{1}_D. \tag{66}$$

It furthermore holds for all causal maps that C and D become independent if we ignore B:

$$\mathrm{Tr}_B\left[\tau_{CBD}\right] = \rho_C \otimes \frac{1}{2}\mathbb{1}_D. \tag{67}$$

The experimental statistics inherit these properties: letting $u(d) \equiv \frac{1}{2}\ \forall d = \pm 1$ denote the uniform probability distribution, we have

$$P(cdb) = pP_{CB}(cb)u(d) + (1-p)P_C(c)P_{BD}(bd). \tag{68}$$

The marginal distributions over c and d in both terms are identical,

$$\sum_b P_{CB}(cb) = P_C(c), \tag{69}$$

$$\sum_b P_{BD}(bd) = u(d), \tag{70}$$

and, if we ignore b, then c and d become independent,

$$\sum_b P(cdb) = P_C(c)u(d). \tag{71}$$

Intuitive simple version of the witness

Suppose that the marginal on C is completely mixed, so that a probabilistic mixture of common-cause and cause-effect takes the form

$$\tau'_{CBD} = p\rho_{CB} \otimes \frac{1}{2}\mathbb{1}_D + (1-p)\frac{1}{2}\mathbb{1}_C \otimes \tau_{BD}, \tag{72}$$

with

$$\mathrm{Tr}_B\rho_{CB} = \frac{1}{2}\mathbb{1}_C, \quad \mathrm{Tr}_B\tau_{BD} = \frac{1}{2}\mathbb{1}_D. \tag{73}$$

Under this assumption, we can construct a witness in terms of the joint probabilities of supplementary equation 56 which is simply the expectation value of τ_{CBD} for a product of Pauli observables,

$$\begin{aligned} \mathcal{C}^0_{CD} &\equiv \sum_{cdb} cdbP(cdb) \\ &= \mathrm{Tr}\left[\tau_{CBD}\sigma_C^s \otimes \sigma_B^u \otimes T_D\left(\sigma_D^t\right)\right], \end{aligned} \tag{74}$$

For any $s,t,u \in 1,2,3$, this is zero for any probabilistic mixture, as can be seen by inserting supplementary equation 72 into supplementary equation 74. Therefore, if one can assume that $\rho_C = \frac{1}{2}\mathbb{1}$, then non-zero value of $\mathcal{C}^0_{CD}$ heralds a physical mixture.

General form of the witness

If we cannot justify the assumption that $\rho_C = \frac{1}{2}\mathbb{1}$, then we must use a more general version of the witness. We will now propose such a witness: a measure of induced correlations that is designed to be zero for probabilistic mixtures even if $\rho_C \neq \frac{1}{2}\mathbb{1}$.

Given the joint distribution $P(cbd)$, one can calculate the marginal $P(b) = \sum_{cd} P(cbd)$ and the conditional distribution $P(cd|b) = P(cbd)/P(b)$. For each value of b, the latter is a distribution over c and d, and therefore the correlations between the two can be quantified by their covariance,

$$\begin{aligned}\mathrm{cov}(c,d|b) &= \sum_{cd} cdP(cd|b) \\ &- \left[\sum_{cd} cP(cd|b)\right]\left[\sum_{cd} dP(cd|b)\right].\end{aligned} \tag{75}$$

We now define our witness to be the weighted difference of the covariances in the conditional distributions,

$$\mathcal{C}_{CD} = 2\sum_{b=\pm 1} bP(b)^2 \mathrm{cov}(cd|b). \tag{76}$$

We will prove that this choice has the desired properties in the following.

Simplification in limiting case

The witness $\mathcal{C}_{CD}$ reduces to $\mathcal{C}^0_{CD}$ if certain marginals of $P(cdb)$ are uniform, specifically, if

$$P(cb) \equiv \sum_d P(cdb) = \frac{1}{4}\ \forall c, b, \tag{77}$$

$$P(db) \equiv \sum_c P(cdb) = \frac{1}{4}\ \forall d, b. \tag{78}$$

This ensures that each b occurs with equal probability, $P(b) = \frac{1}{2}$, and consequently the conditional distributions also satisfy

$$P(c|b) \equiv \sum_d P(cd|b) = \frac{1}{2}\ \forall c, b, \tag{79}$$

$$P(d|b) \equiv \sum_c P(cd|b) = \frac{1}{2}\ \forall d, b, \tag{80}$$

that is, the conditional distribution $P(cd|b)$ has uniform marginals on c and d. The expectation values $\langle c\rangle$ and $\langle d\rangle$ under this distribution are zero, so that the covariance simplifies to

$$\mathrm{cov}(c,d|b) = \langle cd\rangle = \sum_{cd} cdP(cd|b), \tag{81}$$

and therefore

$$\begin{aligned}\mathcal{C}_{CD} &= 2\sum_b bP(b)^2 \sum_{cd} cdP(cd|b) \\ &= \sum_{cdb} cdbP(cdb) \equiv \mathcal{C}^0_{CD}.\end{aligned} \tag{82}$$

In this sense, the witness $\mathcal{C}_{CD}$ is a generalization of the expectation value of the simple product of Paulis that defines $\mathcal{C}^0_{CD}$

Casting $\mathcal{C}_{CD} = 0$ directly in terms of count numbers

In order to facilitate the proof below as well as the assessment of whether or not $\mathcal{C}_{CD} = 0$ based on experimental data, we cast the witness in a different form. To this end, we note that, if c and d are binary variables whose values are labelled ± 1, then their covariance under a conditional distribution $P(cd|b)$ takes the form

$$\mathrm{cov}(c, d|b) = 4\left[P(++|b)P(--|b) - P(+-|b)P(-+|b)\right]. \tag{83}$$

This allows us to rewrite the witness in terms of the joint probabilities $P(cbd)$ as

$$\mathcal{C}_{CD} \equiv 8 \sum_{b=\pm 1} b\left[P(++b)P(--b) - P(+-b)P(-+b)\right]. \tag{84}$$

$\mathcal{C}_{CD} = 0$ for probabilistic mixtures

Now we can show that $\mathcal{C}_{CD}$ is zero for any probabilistic mixture of common-cause and cause-effect relations. Recall that, since b only takes two values, the marginal independence (supplementary equation 71),

$$\sum_b P(cdb) = P_C(c)u(d) = \frac{1}{2}P_C(c) \tag{85}$$

implies that

$$P(cd, -) = \frac{P_C(c)}{2} - P(cd, +). \tag{86}$$

This allows us to rewrite the $b = -1$ term in supplementary equation 84 as

$$\begin{aligned} &P(++,-)P(--,-) - P(+-,-)P(-+,-) \\ &= -\frac{P_C(+)}{2}P(--,+) - \frac{P_C(-)}{2}P(++,+) \\ &+ \frac{P_C(+)}{2}P(-+,+) + \frac{P_C(-)}{2}P(+-,+) \\ &+ [P(++,+)P(--,+) - P(+-,+)P(-+,+)], \end{aligned} \tag{87}$$

hence the witness reduces to

$$\begin{aligned} \mathcal{C}_{CD} &= 4[P_C(-)P(++,+) - P_C(-)P(+-,+) \\ &\quad - P_C(+)P(-+,+) + P_C(+)P(--,+)] \end{aligned} \tag{88}$$

$$= 4\sum_{cd} cd\left[1 - P_C(c)\right]P(cd, +) \tag{89}$$

Our core hypothesis, of a probabilistic mixture, implies that $P(cd, +)$ is a convex combination of two terms, each one a product distribution over cd. Substituting supplementary equation 68 and distributing the sums,

$$\begin{aligned} \mathcal{C}_{CD} &= 4p\left[\sum_c c\left[1 - P_C(c)\right]P_{CB}(c, +)\right]\left[\sum_d d\, u(d)\right] \\ &\quad + 4(1-p)\left[\sum_c c\left[1 - P_C(c)\right]P_C(c)\right]\left[\sum_d P_{BD}(d, +)\right]. \end{aligned} \tag{90}$$

In the first term, we have the average over $d = \pm 1$ under the uniform distribution, which is zero. In the second term, the sum over c gives $P_C(+)P_C(-) - P_C(-)P_C(+) = 0$. Thus, for any causal map that is a probabilistic mixture of cause-effect and common-cause mechanisms of the form of supplementary equation 64, we have

$$\mathcal{C}_{CD} = 0. \tag{91}$$

Measuring the witness from experimental data

We calculate the witness $\mathcal{C}_{CD}$ explicitly from experimental count numbers $\tilde{P}(c,d,b)$ using supplementary equation 84,

$$\mathcal{C}_{CD} = \frac{\sum_{b=\pm 1} b\left(\tilde{P}(++b)\tilde{P}(--b) - \tilde{P}(+-b)\tilde{P}(-+b)\right)}{\left(\sum_{c,d,b=\pm 1} \tilde{P}(c,d,b)\right)^2}. \tag{92}$$

The uncertainty on the witness is calculated by assuming Poissonian noise on the count numbers and propagating the errors through supplementary equation 92.

Supplementary Note 6: Bounds on induced mutual information in Berkson's paradox

In the following, we derive an upper bound on the mutual information between two causes, D and E, conditioned on their common effect, B, under the assumption that the two influences are combined probabilistically, that is,

$$P(B|DE) = (1-p)P_{\mathcal{D}}(B|D) + pP_{\mathcal{E}}(B|E). \tag{93}$$

The derivation is cast in terms of classical variables, but an extension to the quantum case is given at the end.

The distribution over DE conditional on some value of B can be obtained by Bayesian inversion. Note that, since D and E do not share a common cause, our prior probability distribution over them takes the form of a product of two generic probability distributions, which we denote by $Q(D)$ and $Q(E)$. It follows that

$$\begin{aligned} P(DE|B) &\equiv P(B|DE)P(DE)/P(B) \\ &= (1-p)Q(E)\frac{P_{\mathcal{D}}(B|D)Q(D)}{P(B)} \\ &+ p\,Q(D)\frac{P_{\mathcal{E}}(B|E)Q(E)}{P(B)}, \end{aligned} \tag{94}$$

where $P(B) \equiv \sum_{DE} P(B|DE)Q(D)Q(E)$. For each value b of B, the fractions are distributions over D and E, respectively, but not necessarily normalized. Let $P^b_{\mathcal{D}}(D)$ and $P^b_{\mathcal{E}}(E)$ denote the corresponding normalized distributions, introducing the b-dependent modified weight q^b to absorb the difference in normalization:

$$q^b = p\frac{1}{P(B=b)}\sum_E P_{\mathcal{E}}(B=b|E)Q(E), \tag{95}$$

or, equivalently,

$$(1-q^b) = (1-p)\frac{1}{P(B=b)}\sum_D P_{\mathcal{D}}(B=b|D)Q(D), \tag{96}$$

and

$$P^b_{\mathcal{E}}(E) = \frac{p}{q^b}\frac{1}{P(B=b)}P_{\mathcal{E}}(B=b|E)Q(E) \tag{97}$$

$$P^b_{\mathcal{D}}(D) = \frac{1-p}{1-q^b}\frac{1}{P(B=b)}P_{\mathcal{D}}(B=b|D)Q(D). \tag{98}$$

For the purpose of this derivation, we will focus on a single value b and, for brevity, suppress the explicit b-dependence in the following. In this new notation,

$$P(DE) = (1-q)Q(E)P_{\mathcal{D}}(D) + qQ(D)P_{\mathcal{E}}(E). \tag{99}$$

We will show that the mutual information $I(D:E)$ in this distribution is maximal if $P_{\mathcal{D}}(D)$ and $P_{\mathcal{E}}(E)$ each produce a single value with certainty. To see this, consider the mutual information as a functional of two arguments, the marginal distribution over E,

$$P(E) = (1-q)Q(E) + qP_{\mathcal{E}}(E), \tag{100}$$

and the conditional

$$\begin{aligned} P(D|E) = &\frac{(1-q)Q(E)}{(1-q)Q(E)+qP_{\mathcal{E}}(E)}P_{\mathcal{D}}(D) \\ &+ \frac{qP_{\mathcal{E}}(E)}{(1-q)Q(E)+qP_{E}(E)}Q(D). \end{aligned} \tag{101}$$

One can show (Ref. [6], theorem 2.7.4) that the mutual information is convex in the second argument, that is, for a fixed marginal $P(E)$,

$$\begin{aligned} I(D:E)&\left[P(E), \lambda P^0(D|E) + (1-\lambda)P^1(D|E)\right] \\ &\leq \lambda I(D:E)\left[P(E), P^0(D|E)\right] \\ &+ (1-\lambda) I(D:E)\left[P(E), P^1(D|E)\right]. \end{aligned} \tag{102}$$

In order to apply this fact to our problem, suppose that we fix the marginal $P(E)$ – and consequently the fractions in the expression for $P(D|E)$ above – but take a convex combination

$$P_{\mathcal{D}}(D) = \lambda P^0_{\mathcal{D}}(D) + (1-\lambda)P^1_{\mathcal{D}}(D), \tag{103}$$

so that the resulting $P(D|E)$ is a convex combination with weight λ as well. In this case, an upper bound on the mutual information follows. It follows that, for fixed $P_{\mathcal{E}}(E)$ and q, the largest mutual information is achieved when the distribution $P_{\mathcal{D}}(D)$ is extremal, meaning that it produces one value with certainty. We express this as $P_{\mathcal{D}}(D) = \delta(D)$ for short. We do not specify which value of D is found with certainty, since the mutual information depends only on the probabilities of different values, but not on their labels. By symmetry, in order to maximize the mutual information we must also have $P_{\mathcal{E}}(E) = \delta(E)$.

The maximal mutual information between D and E for a distribution constrained to the form (99) is therefore achieved by a distribution of the form

$$P(DE) = (1-q)Q(E)\delta(D) + qQ(D)\delta(E). \tag{104}$$

In order to evaluate the maximal mutual information explicitly, we make two simplifying assumptions: first, let us assume that the prior distributions $Q(D)$ and $Q(E)$ are both uniform, that is, that we have no additional information about them beyond what we can retrodict from B. Let us furthermore assume that D and E range over an equal number of values, N. Symmetry then suggests that the mutual information is maximal when $q = \frac{1}{2}$, which can be verified analytically. In this case, we obtain

$$I(D:E) = \log N - \frac{N+1}{N}\left[\log(N+1) - 1\right] \tag{105}$$

with log denoting the logarithm used to calculate the entropy. By contrast, the maximal mutual information between D and E without any constraints is $\log N$. If D and E are bits ($N=2$) and we calculate the logarithms in base 2, the upper bound on the mutual information becomes

$$I(D:E) \leq \frac{5}{2} - \frac{3}{2}\log_2(3) \approx .12. \tag{106}$$

Now consider the case where D and E are quantum systems. Their state under post-selection on a measurement outcome b on B can be written

$$\rho^b_{DE} = (1-q)\rho_D \otimes \frac{\mathbb{1}_E}{2} + q\frac{\mathbb{1}_D}{2} \otimes \rho_E. \tag{107}$$

As in the classical case, we consider the prior over D and E to be uniform, that is, the maximally mixed quantum state. This implies that there is in fact only one non-trivial density operator on D (in the first term) and E (in the second) in the entire problem. Consequently there exist preferred bases of $\mathcal{H}_D$ and $\mathcal{H}_E$, namely the eigenbases of ρ_D and ρ_E, in which all density operators of interest are diagonal and thus effectively reduced to classical probability distributions. Therefore the results from the classical case carry over, and we recover the upper bound above as a function of the dimension of the Hilbert spaces $N = \dim \mathcal{H}_D = \dim \mathcal{H}_E$.

SUPPLEMENTARY DISCUSSION

Supplementary Discussion 1: Related work on superpositions of causal orders

We here discuss related work that considers the question of whether one can prepare a quantum-coherent mixture of different causal orders [7, 8]. For a pair of quantum systems, A and B, the idea is to prepare a quantum-coherent mixture of A being the cause of B and of B being the cause of A. By contrast, in this article we seek only to prepare a quantum-coherent mixture of A being a cause of B and of A and B having a common cause. There is an important difference between the two objectives. In our case, A and B can be time-like separated, with A to the past of B. In the case of a quantum-coherent mixture of causal orders, on the other hand, the temporal order is different in the two terms of the mixture and consequently these must be embedded differently in space-time. This is the sense in which achieving a quantum-coherent mixture of causal orders requires one to abandon the assumption of a pre-defined global causal structure.

Nonetheless, the approach of defining probabilistic, physical, and quantum-coherent mixtures of causal relations that is espoused in the present article can be applied to the case of two cause-effect relations, in particular, A causing B and B causing A, and it is interesting to see what lessons are learned from doing so. We begin with the classical case.

In our approach, the overall causal structure of a given scenario is depicted by a directed acyclic graph (DAG). If one considers a probabilistic mixture of causal relations, then one must include enough causal influences in the graph to accommodate the causal relations that hold in any given element of the mixture. The DAG associated to a probabilistic mixture of cause-effect and common-cause, depicted in Supplementary Fig. 1(b), therefore includes both a cause-effect pathway and a common-cause pathway between A and B. Similarly, it follows that the graph associated to a probabilistic mixture of A causing B and B causing A must have both a pathway wherein A causes B and another wherein B causes A.

Furthermore, as noted previously, in order to physically realize a probabilistic mixture of different causal relations, one requires a switch variable J that can influence one or more variables in the system and modify how they causally depend on other variables. For a probabilistic mixture of cause-effect and common-cause relations, it was shown in Supplementary Note 1 that this switch variable must influence B alone. But what does it imply for a probabilistic mixture of A causing B and B causing A? In this case, the switch variable (call it J) cannot influence B alone, but must instead influence both A and B. This is because as one varies between $J = 0$ and $J = 1$, A must toggle between having a causal dependence on B and not having such a dependence while B must simultaneously toggle between not having a causal dependence on A and having such a dependence. As such, the switch variable defines a common-cause pathway between A and B. The overall causal structure is depicted in Supplementary Fig. 3.

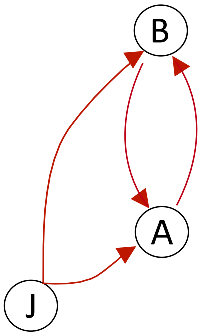

Supplementary Fig. 3. **Mixture of causal orders** In order to physically realize a probabilistic mixture of different causal orders, one requires a switch variable J that can influence both variables A and B. One also requires a cycle in the causal structure.

One can immediately observe two uncomfortable facts about the overall causal structure.

First, the natural constraint on physical realizability of probabilistic mixtures articulated in Supplementary Note 1 has been violated. The objective was to have a probabilistic mixture of causal relations between A and B every element of which was purely cause-effect (either A causing B or B causing A). However, any attempt to physically realize such a mixture introduces a causal relation that is not purely cause-effect, namely, the common cause J.

Second, and more importantly, one notes that the overall causal structure is not a directed acyclic graph because it includes a cycle. It is unclear how to make sense of such graph. One can no longer interpret the causal relations therein

using the interventionist notion of causation that is standard for directed acyclic graphs. The reason is as follows. The interventionist notion of causation presumes that causal mechanisms in the graph are autonomous: the mechanism that describes how one variable in the graph is causally influenced by its parents can be varied independently of the mechanism that describes how any other variable in the graph is causally influenced by its parents. But this assumption of autonomy cannot be maintained in graphs with cycles. For instance, consider a graph having a cycle between a pair of binary variables, A and B. If the two causal mechanisms were autonomous, then it ought to be possible to take them to be $A := B$ and $B := A \oplus 1$ respectively. But the latter pair of mechanisms yields a contradiction, so the mechanisms cannot be varied independently of one another.

The second of these concerns may be surmountable in the case of a probabilistic mixture of A causing B and of B causing A, since only one of the two pathways is active for a given value of the switch variable J.

If, however, one considers instead a physical mixture of A causing B and of B causing A, then both pathways must be active simultaneously, and there is no way to deny the necessity of the cycle.

The conceptual problems introduced by the presence of cycles in the causal graph persist if one replaces classical variables with quantum systems. In the approach we propose in this article, a quantum-coherent mixture of causal relations between quantum systems is necessarily a physical mixture of those causal relations. Consequently, a quantum-coherent mixture of causal orders in our approach requires a graph with a cycle, with all the interpretive ambiguity that this entails.

Finally, even if one can make sense of graphs with cycles, it remains unclear how one could ever hope to realize these experimentally because in the context of relativity theory, a causal cycle is a closed time-like curve which one expects is only physically realizable in very exotic physical scenarios. In our approach, therefore, realizing a quantum-coherent mixture of causal orders, if it is possible at all, is likely to only be possible in very exotic scenarios.

Some recent work by Procopio *et al.* [9] claims to achieve an experimental realization of a superposition of causal orders in a tabletop quantum optics experiment. This seems to contradict our claim that one is likely to require exotic physics to achieve such a thing. We therefore turn to the details of this experiment and why we do not believe that it can be accurately described as achieving a superposition of causal orders.

The objective is to realize, in a quantum optical setting, the quantum switch proposed by Chiribella *et al.* [10] and explored in Ref. [7], wherein the order of two gates is controlled by an ancillary quantum system that is prepared and post-selected in a superposition of the states which prescribe a definite causal order. This has been proposed as a means of achieving a superposition of causal orders. The experiment is based on a folded Mach-Zehnder interferometer whereby the order of two gates, call them U and V, is determined by the path taken by the photon. Due to the particular geometry, one requires a version of the U and V gate in each path of the interferometer.

This set-up is optically equivalent to an unfolded interferometer. In the latter case, it is clear that one requires a version of the U and V gate in each path of the interferometer, call them U_1, V_1 and U_2, V_2 respectively. The different orders that one switches between are: U_1 is implemented and then V_2 is implemented, and U_2 is implemented and then V_1 is implemented. The situation is clearly not one wherein one toggles between a photon passing through two fixed spatio-temporal regions in one of two different orders.

For the case of the folded interferometer used in the experiment, it is still the case that one requires two versions of each gate; it is simply that the two versions correspond to the gate functioning at different times. Call the early versions of the two gates U_1, V_1 and the late versions U_2, V_2. Again, the different orders that one switches between are: U_1 is implemented and then V_2 is implemented, and U_2 is implemented and then V_1 is implemented.

If one instead required that each gate act only once in a localized spatio-temporal region – for instance, by putting shutters that let a photon through the gate only in a narrow window of time – then the experimental set-up in question would no longer realize a quantum switch.

SUPPLEMENTARY REFERENCES

[1] Choi, M. D. Completely positive linear maps on complex matrices. *Linear Algebra Appl.* **10**, 285–290 (1975).
[2] Jamiołkowski, A. Linear transformations which preserve trace and positive semidefiniteness of operators. *Rep. Math. Phys.* **3**, 275–278 (1972).
[3] Leifer, M. & Spekkens, R. W. Towards a formulation of quantum theory as a causally neutral theory of bayesian inference. *Phys. Rev. A* **88**, 052130 (2013).
[4] Ried, K. *et al.* A quantum advantage for inferring causal structure. *Nat. Phys.* **11**, 414–420 (2015).
[5] James, D. F. V., Kwiat, P. G., Munro, W. J. & White, A. G. Measurement of qubits. *Physical Review A* **64**, 052312 (2001).
[6] Cover, T. M. & Thomas, J. A. *Elements of information theory* (Wiley, 2006).
[7] Chiribella, G. Perfect discrimination of no-signalling channels via quantum superposition of causal structures. *Phys. Rev. A* **86**, 040301 (2012).
[8] Oreshkov, O., Costa, F. & Brukner, C. Quantum correlations with no causal order. *Nat. Commun.* **3**, 1092 (2012).

[9] Procopio, L. M. *et al.* Experimental superposition of orders of quantum gates. *Nat. Commun.* **6**, 7913 (2015).
[10] Chiribella, G., D'Ariano, G. M., Perinotti, P. & Valiron, B. Quantum computations without definite causal structure. *Phys. Rev. A* **88**, 022318 (2013).
